# ChatGPT and mycosis– a new weapon in the knowledge battlefield

**DOI:** 10.1186/s12879-023-08724-9

**Published:** 2023-10-27

**Authors:** Yi Jin, Hua Liu, Bin Zhao, Weihua Pan

**Affiliations:** 1 Department of Dermatology, Shanghai Key Laboratory of Medical Mycology, Second Affiliated Hospital of Naval Medical University, Shanghai, 200003 P.R. China; 2 Department of Anesthesiology, Shanghai Ninth People’s Hospital, Shanghai Jiao Tong University School of Medicine, Shanghai, China; 3 Department of Anesthesiology and SICU, Xinhua Hospital, School of Medicine, Shanghai Jiao Tong University, Shanghai, 200092 P.R. China

**Keywords:** ChatGPT, Mycosis, Applications

## Abstract

As current trend for physician tools, ChatGPT can sift through massive amounts of information and solve problems through easy-to-understand conversations, ultimately improving efficiency. Mycosis is currently facing great challenges, including high fungal burdens, high mortality, limited choice of antifungal drugs and increasing drug resistance. To address these challenges, We asked ChatGPT for fungal infection scenario-based questions and assessed its appropriateness, consistency, and potential pitfalls. We concluded ChatGPT can provide compelling responses to most prompts, including diagnosis, recommendations for examination, treatment and rational drug use. Moreover, we summarized exciting future applications in mycosis, such as clinical work, scientific research, education and healthcare. However, the largest barriers to implementation are deficits in indiviudal advice, timely literature updates, consistency, accuracy and data safety. To fully embrace the opportunity, we need to address these barriers and manage the risks. We expect that ChatGPT will become a new weapon in in the battlefield of mycosis.

## Introduction

ChatGPT (Chat Generative Pre-trained Transformer) is a large language model (LLM) developed by OpenAI launched in November 2022. It mimics natural language and solves cognitive problems by reinforcing learning from online resources using human feedback. ChatGPT has medical licensing examination performance as an undergraduate third-year medical student, and has, therefore, stimulated urgent discussions within medicine [1]. Text is actually a projection of the world, as well as of medicine and science. ChatGPT has rapidly become the current trend for the past months, because of its endless applications in medicine such as analysing vast amounts of medical data and as summarization tool, aiding in ‘standardized’ clinic letters and clinical trials, helping diagnosis and treatment, facilitating scientific research, improving medical education and communication with patients [2, 3]. In future, ChatGPT may also become a new weapon in the knowledge battlefield in mycosis.

### Problems in mycosis: high burdens and high mortality

Despite the broad importance and socio-economic impact of medical mycology, research on fungal infections has lagged behind compared to other pathogens. The recent SARS-CoV-2 pandemic has highlighted the importance of fungal infections for morbidity and mortality [4]. In some studies, a two- to tenfold higher incidence of candidemia has been reported in patients with COVID-19 compared with patients without COVID-19 [5, 6]. Candida auris( *C. auris*) has become a global fungal public health threat. COVID-19-associated *C. auris* outbreaks have resulted in mortality rates ranging from 30% [7] to 83% [8] in those with candidemia. The incidence of mucormycosis has increased dramatically compared to pre-COVID-19. COVID-19-associated rhinoorbital mucormycosis (ROM) has mortality rates of 14% and higher [9], whereas pulmonary or disseminated mucormycosis has mortality rates in excess of 80% [10]. In the largest multicentre studies, COVID-19-associated pulmonary aspergillosis (CAPA) is an independent prognostic factor for mortality, with hazard ratios between 1.45 and 1.97 [11, 12]. CAPA has been consistently associated with COVID-19 mortality rates of more than 50% [13–16]. New hosts contributing to new therapies for malignant and autoimmune disease have increased [17, 18]; and new patterns, including aspergillosis in pulmonary tuberculosis (PTB) and chronic obstructive pulmonary disease (COPD), are emerging [19, 20]. High fungal burdens, high mortality, limited choice of antifungal drugs and increasing drug resistance caused a huge impact on public health, which underscore the urgent need for increasing awareness of fungal infection, building prompt and accurate diagnostic and therapeutic capacity, and solving multidrug resistance. ChatGPT, as a tool for doctors, can be used to improve efficiency and reduce workload, and applied to clinical work, scientific research, education and healthcare in mycosis.

### Conversation with ChatGPT regarding mycosis

We asked ChatGPT for fungal infection scenario- based questions, then assessed the appropriateness, consistency, pitfalls of the AI(artificial intelligence)tool.

**Fig. 1:**
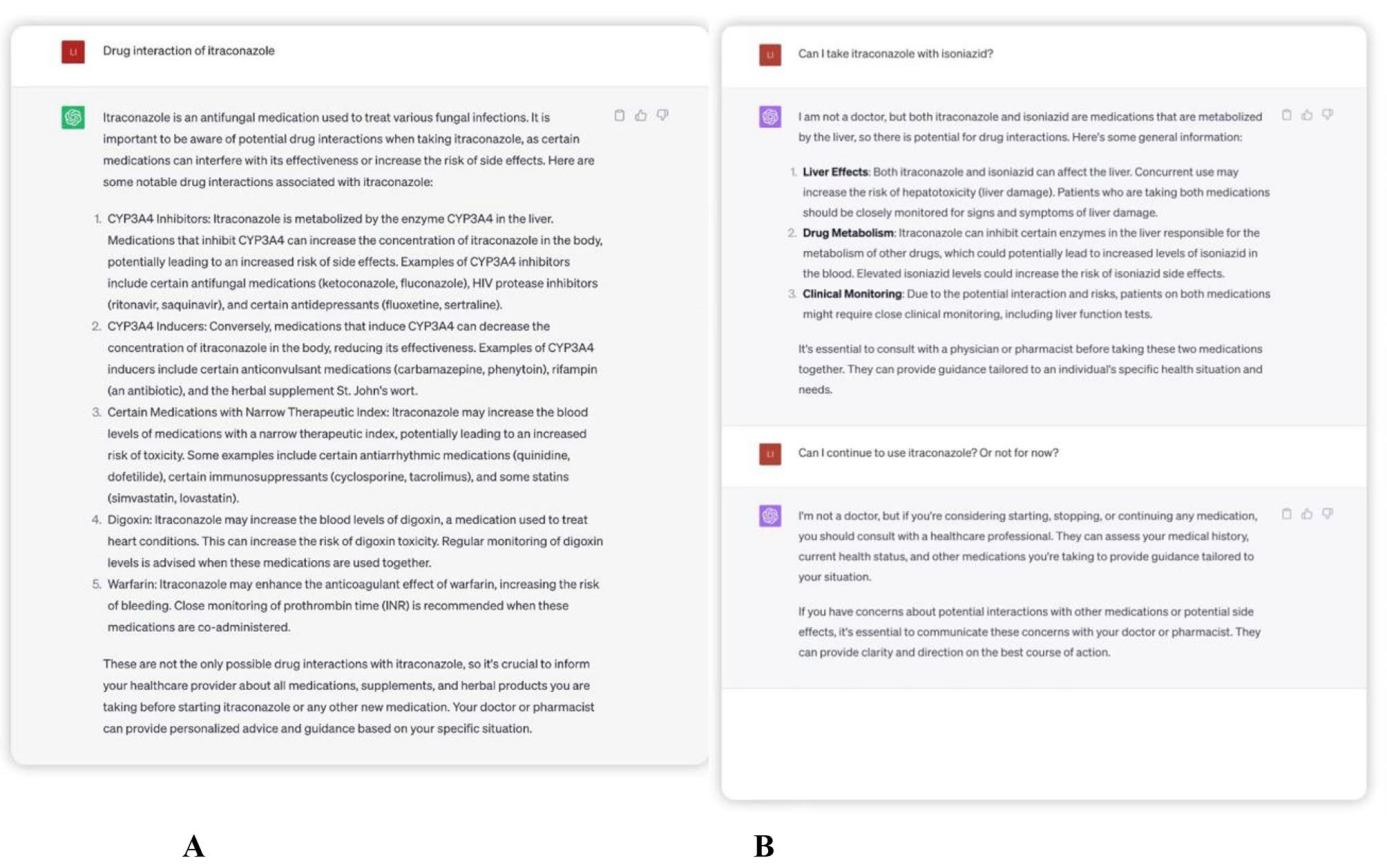
Screenshots examples of tasks ChatGTP completed. **A.** Conversation with ChatGPT about drug interaction of itraconazole. **B.** Suggestions of ChatGPT for rational drug use. General advice is provided rather than specific guidance on rational use of drugs, and there is no clear time window for drug prohibition

First, we had a conversation with ChatGPT about drug interaction of itraconazole and asked suggestions for rational drug use (Fig. 1). We found general advice is provided rather than specific guidance on rational use of drugs, and there was no clear time window for drug prohibition.

**Fig. 2:**
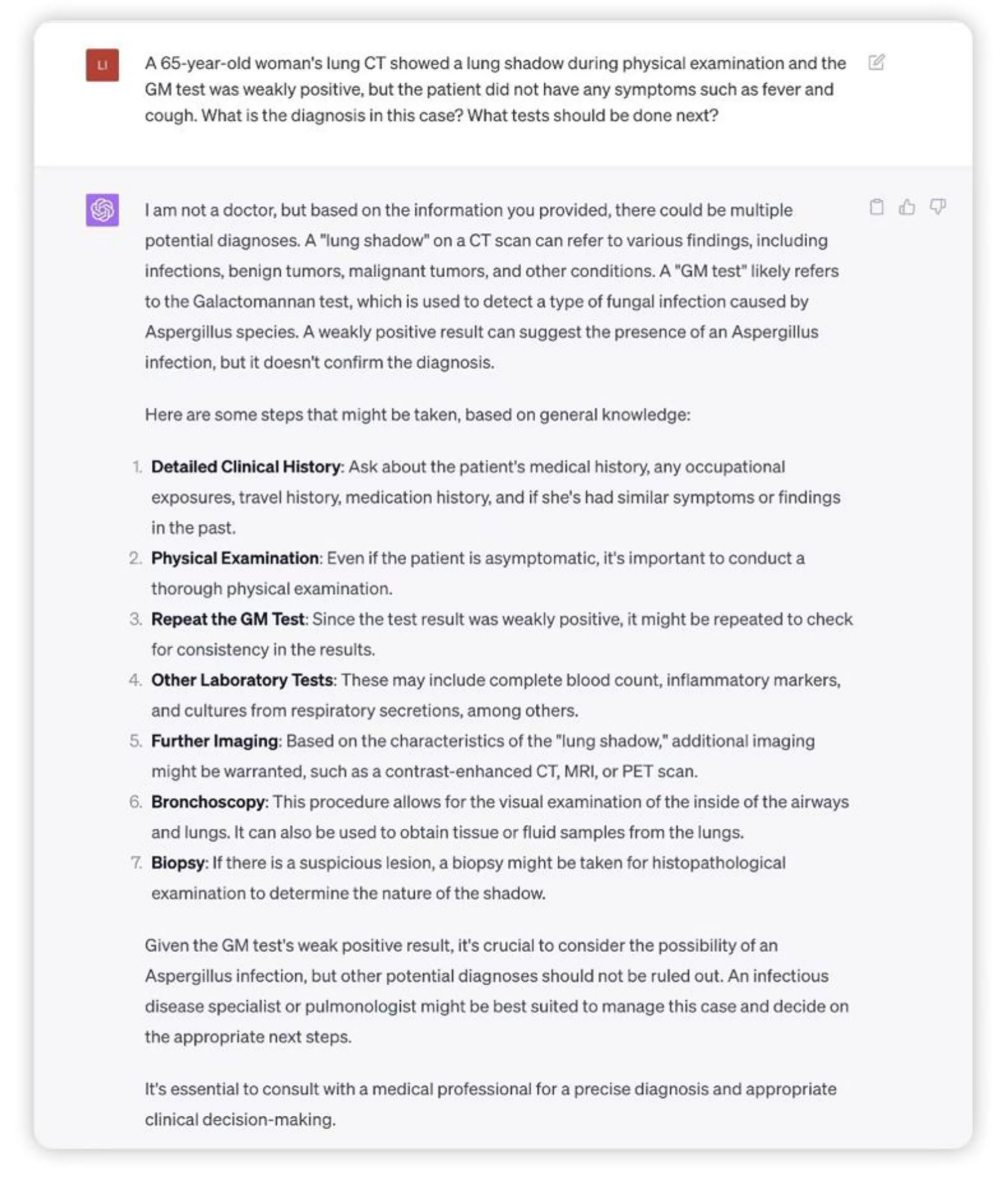
Regarding a case of lung shadow and GM test weak positive, the diagnosis and recommendations for further examination are appropriately described

Second, We used ChatGPT to input examinations such as lung shadow and GM test weak positive, and it was able to analyse the data and provide a probable diagnosis and a rational list of recommendations for further examination for the specific condition (Fig. 2).

**Fig. 3:**
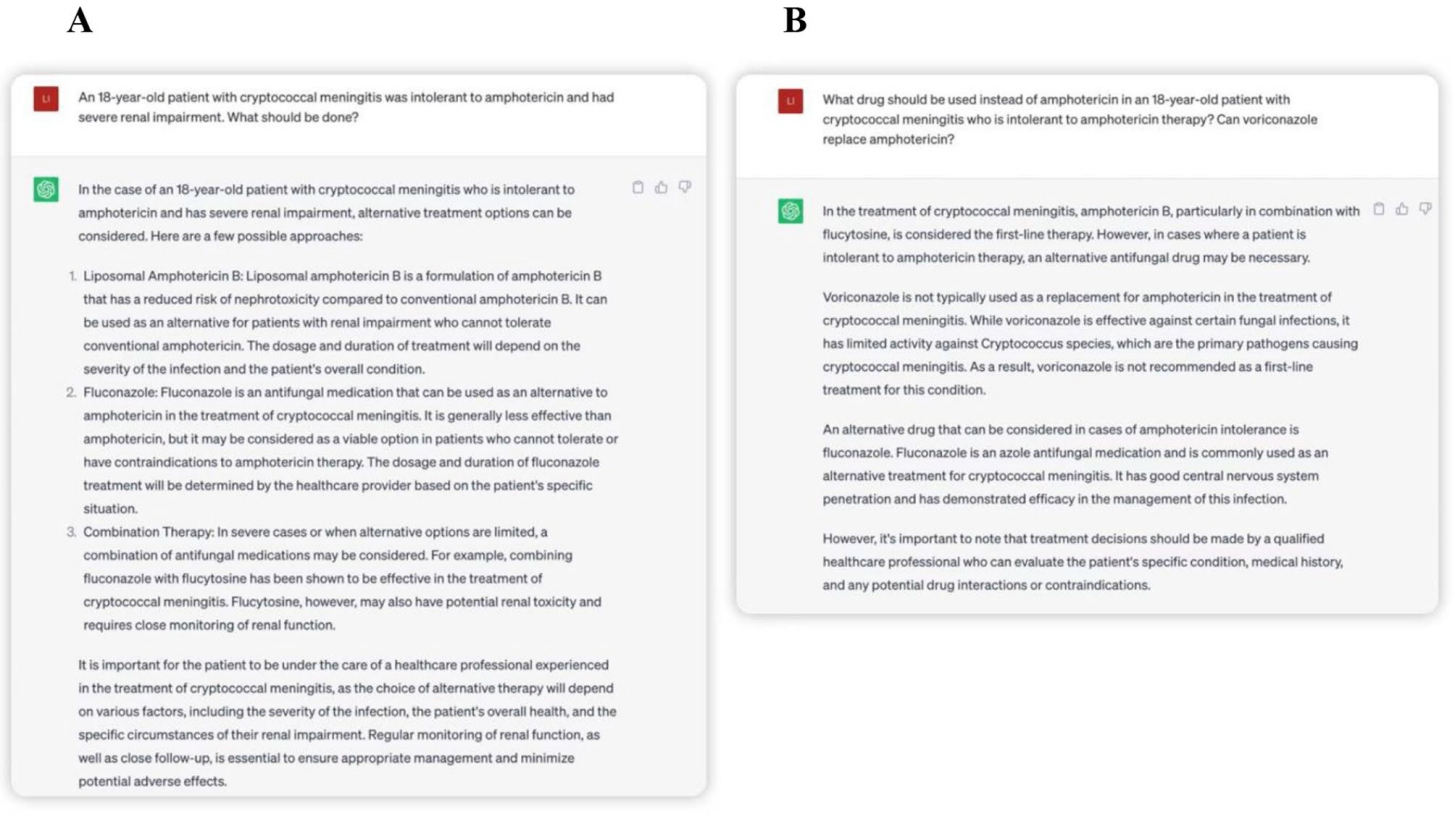
Conversation about a patient with cryptococcal meningitis who is intolerant to amphotericin. (**A**) ChatGTP provides correct suggestions for alternatives for induction therapy. (**B**) ChatGPT did not mention Liposomal Amphotericin B for alternative therapy and lack of updating literature of voriconazole. For complex clinical questions, the literature cannot be updated in time, and the answers are not consistent

Third, we had a conversation about a patient with cryptococcal meningitis who was intolerant to amphotericin twice. For the first time, ChatGTP provided correct suggestions for alternatives for induction therapy. However, for the second time, ChatGPT did not mention Liposomal Amphotericin B for alternative therapy. Futhermore, ChatGPT lackd literature updates on voriconazole. We found that, for complex clinical questions, the answers of ChatGPT lackd consistency and coherence, and the literature could not be updated in time (Fig. 3).

**Fig. 4:**
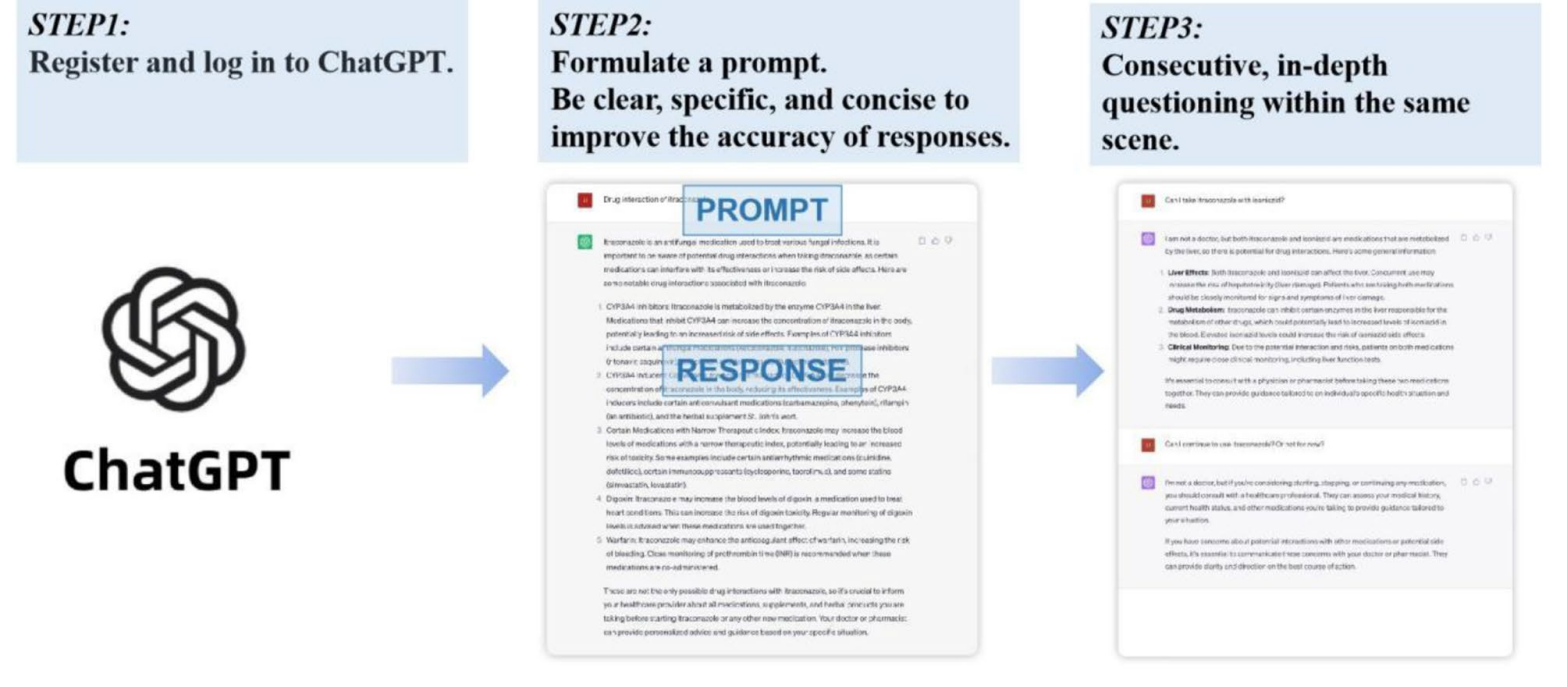
Step-by-step workflow diagram for applying GPT. Step1 is to register and log in to GPT. Step2 is to formulate a prompt. Please note that for ensuring accurate and high-quality responses, instructions should be clear and specific, and concise. Step3 allows for continuous and in-depth questioning on the same topic to ensure completeness and depth of the responses

We conclude ChatGPT provides compelling responses to most prompts, including diagnosis, recommendations for further examination, appropriate treatment and suggestions for rational drug use. The largest barriers to the implementation of ChatGPT in clinical practice are deficits in situational awareness, inference, and consistency. These limitations have the potential to compromise patient safety. While ChatGPT can provide general directions, it lacks the ability to offer individualized medication recommendations. Hence, it is imperative to recognize and uphold the indispensable role of doctors in patient treatment——a responsibility that cannot be replaced by AI. As LLMs evolve rapdily and incorporate more information, it is crucial that clinicians become familiar with and apply the new technology in mycosis (Fig. 4), thus driving the progress of mycology.

### Applications of ChatGPT in mycosis and future direction

**Fig. 5:**
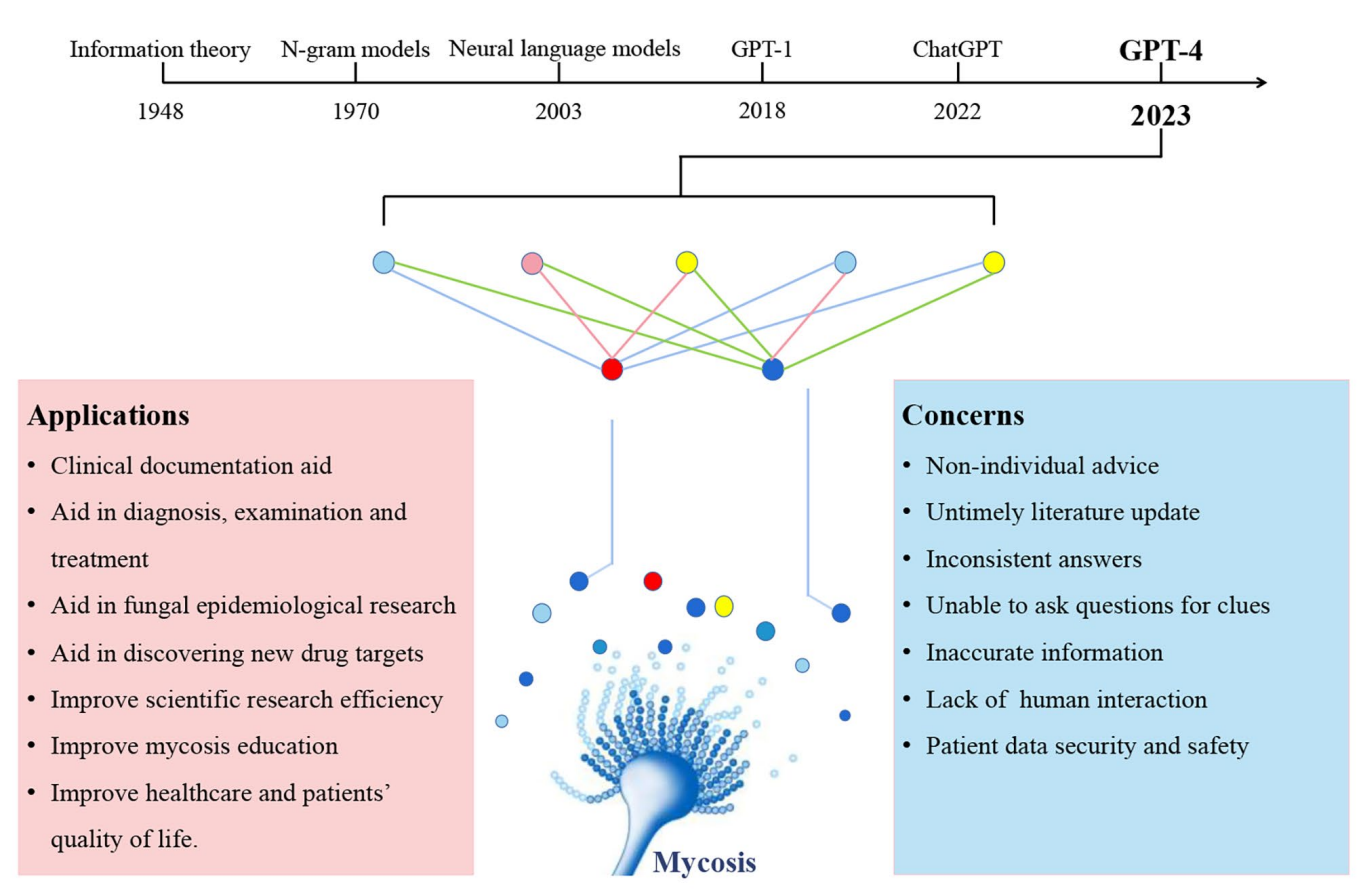
History of large language model, applications and concerns of GPT4

We summarized exciting future applications and concerns in mycosis(Figure5).The strength of ChatGPT is the ability to sift through massive amounts of information and produce responses in a manner that is conversational and easy to understand [21]. ChatGPT provides a basis for more flexible and efficient fungal epidemiological research. Indeed, epidemiological research also relies on efficient and reliable data collection, recording and analysis. ChatGPT not only solves the difficulty of remote inquiries, but also helps to reduce labour requirements to complete the work [22]. Besides, ChatGPT is usually more accurate and faster than manual statistics and records. It can be seen that computation can help practitioners save time and do more research.

Due to the lack of available antifungal drugs, increasing drug resistance and high mortality, it is urgent to explore the pathogenesis of mycosis and develop new drugs. ChatGPT may serve as a new tool for discovering potential drug targets. ChatGPT can be applied to the ‘language’ of DNA or protein sequences, derive the right relationships between molecules on its own, and predict new proteins which might make good drug targets. Hence, ChatGPT is arguably a more direct line to drug discovery and drug development [23]. The competition and workload in academia increseases. ChatGPT provides opportunities to complete tasks quickly. Therefore, results can be published faster, freeing academics up to focus on new experimental designs [24, 25]. This could significantly accelerate innovation and potentially lead to breakthroughs in mycosis.

The high motality of invasive mycosis is also associated with a lack of awareness of mycosis among clinicians, therefore, strengthening training and education is critical. Learning is best when a knowledgeable and inspiring teacher works with the trainee, enabling them to learn at their own pace and style. ChatGPT, if used correctly, can make processes such as curriculum design, knowledge test and continuing medical education more dynamic than they are currently [26]. This use will help popularize fungal knowledge, enable learners to increase awareness of mycosis, facilitate learning and reflecting.

To popularize the knowledge of mycosis to patients is the key to reduce the incidence of mycosis, improve the compliance of patients and ensure the success of treatment. ChatGPT may be able to enhance healthcare delivery and patients’ quality of life. In fact, patients are often not aware of resources to obtain accurate and personalized information about their condition. A survey reported that around one-third of the US adults sought medical advice on the internet for self-diagnoses, with only around half of these respondents subsequently consulting a physician about the web-based results [27]. People tend to more naturally trust something that mimics human behaviors and responses, such as the responses generated by ChatGPT. The conversational dialogue is more comprehensible than professional guidelines or primary literature. Compared to web surfing, ChatGPT allows you to quickly receive well-tailored answers to the desired questions and receive AI-based medical decision making based on the latest research and guidelines. Even without reviewing the guidelines and papers one by one, you can easily check the information summarized and extracted by AI [28]. Within a constructive and alert regulatory environment, ChatGPT could have a transformative impact in healthcare, augmenting rather than replacing human expertise, and ultimately improving quality of life for many patients.

ChatGPT is not foolproof. Our concerns and urgent problems are as follows: ChatGPT can provide general advice rather than individual suggestions. It cannot ask questions to seek further clues, and sometimes could provide dangerous advice about the anti-microbial contraindications and often miss clinical patient safety cues [29]. Besides, lack of timely updating of literature and human supervision, can output inaccurate and inconsistent information. Furthermore, currently, ChatGPT may not always respect copyright, which is a notable issue to consider. Despite these limitations, ChatGPT’s utilization value can be very high due to its ability to provide personalized interaction and quick response time. To improve the validity and reliability of ChatGPT, it is essential to utilize ChatGPT under the supervision of qualified healthcare professionals. Careful scrutiny and revision of the initial drafts provided by ChatGPT are imperative. Furthermore, selectively select a clinical mycosis data source and feed that information into a ChatGPT conversation frequently, will bridge the case-specific and machine-specific gap, thereby enriching ChatGPT’s knowledge with more customized data. Continual efforts must be made to advance and optimize the capabilities of ChatGPT to ensure its responsible application in the field of mycology. It is crucial to strike a balance between AI integration and clinical judgment, acknowledging the limitations and potential risks associated with overreliance on ChatGPT. Researchers must know how to use the technology judiciously. Therefore, The successful utilization of AI technologies, such as ChatGPT, holds substantial promise for propelling advancements in mycology.

## Summary

ChatGPT plays an important role, but as a tool for the people posing the hypotheses, designing the experiments, and making sense of the results. Ultimately the product must come from—and be expressed by—the wonderful computer in our heads [30]. In the field of mycosis, the focus should be on embracing the opportunity and managing the risks. As ChatGPT evolves and incorporates more information rapidly, rather than AI replacing humans (clinicians), we see it as “clinicians using AI” replacing “clinicians who do not use AI” in the coming years. We expect that ChatGPT will become a new weapon in in the battlefield of mycosis. We should begin to utilize and anticipate the possible misuse of this new technology. We are confident that ChatGPT will support and promote scientific progress, achieving curiosity, imagination, and discovery.

## Data Availability

The datasets analyzed during the current study are available from the corresponding author on reasonable request.

## References

[1] Gilson A, Safranek CW, Huang T (2023). How does ChatGPT perform on the United States medical licensing examination?The implications of large language models for medical education and knowledge assessment. JMIR Med Educ.

[2] Kluger N (2023). Potential applications of ChatGPT in dermatology. J Eur Acad Dermatol Venereol.

[3] Ali SR, Dobbs TD, Hutchings HA, Whitaker IS (2023). Using ChatGPT to write patient clinic letters. Lancet Digit Health.

[4] Hoenigl M, Seidel D, Sprute R (2022). COVID-19-associated fungal Infections. Nat Microbiol.

[5] Kayaaslan B (2021). Characteristics of candidemia in COVID-19 patients; increased incidence, earlier occurrence and higher mortality rates compared to non-COVID-19 patients. Mycoses.

[6] Riche CVW, Cassol R, Pasqualotto AC (2020). Is the frequency of candidemia increasing in COVID-19 patients receiving corticosteroids?. J Fungi.

[7] Mulet Bayona JV (2021). Impact of the SARS-CoV-2 pandemic in candidaemia, invasive aspergillosis and antifungal consumption in a tertiary hospital. J Fungi.

[8] Villanueva-Lozano H (2021). Outbreak of *Candida Auris* Infection in a COVID-19 hospital in Mexico. Clin Microbiol Infect.

[9] Sen M (2021). Epidemiology, clinical profile, management, and outcome of COVID-19-associated rhino-orbital-cerebral mucormycosis in 2826 patients in India—collaborative OPAI-IJO study on Mucormycosis in COVID-19 (COSMIC), report 1. Indian J Ophthalmol.

[10] Hoenigl M (2022). The emergence of COVID-19 associated mucormycosis: a review of cases from 18 countries. Lancet Microbe.

[11] Prattes J (2021). Risk factors and outcome of pulmonary aspergillosis in critically ill coronavirus Disease 2019 patients—a multinational observational study by the European Confederation of Medical Mycology. Clin Microbiol Infect.

[12] Gangneux J-P (2022). Fungal Infections in mechanically ventilated patients with COVID-19 during the first wave: the French multicentre MYCOVID study. Lancet Respir Med.

[13] Salmanton-García J (2021). COVID-19-associated pulmonary aspergillosis, March–August 2020. Emerg Infect Dis.

[14] Janssen NAF (2021). Multinational observational cohort study of COVID-19-associated pulmonary aspergillosis. Emerg Infect Dis.

[15] White PL (2020). A national strategy to diagnose coronavirus Disease 2019-associated invasive fungal Disease in the intensive care unit. Clin Infect Dis.

[16] Bartoletti M (2020). Epidemiology of invasive pulmonary aspergillosis among intubated patients with COVID-19: a prospective study. Clin Infect Dis.

[17] The Lancet (2018). GLOBOCAN 2018: counting the toll of cancer. Lancet.

[18] Hung ML, Liao HT, Chen WS, Chen MH, Lai CC, Tsai CY (2018). Invasive aspergillosis in patients with systemic Lupus Erythematosus: a retrospective study on clinical characteristics and risk factors for mortality. Lupus.

[19] Chinese State Statistical Bureau. China Statistical Yearbook 2018 [cited 2019 Oct 7]. http://www.stats.gov.cn/tjsj/ndsj/2018/indexch.htm.

[20] Wang C, Xu J, Yang L, Xu Y, Zhang X, Bai C, China Pulmonary Health Study Group (2018). Prevalence and risk factors of Chronic Obstructive Pulmonary Disease in China (the China Pulmonary Health [CPH] study): a national cross-sectional study. Lancet.

[21] Yeo YH, Samaan JS, Ng WH (2023). Assessing the performance of ChatGPT in answering questions regarding Cirrhosis and hepatocellular carcinoma [published online ahead of print, 2023 Mar 22]. Clin Mol Hepatol.

[22] Wang Y, Yuan M, Lv H, Peng J, Wilson IA, Wu NC (2022). A large-scale systematic survey reveals recurring molecular features of public antibody responses to SARS-CoV-2. Immunity.

[23] Savage N (2023). Drug discovery companies are customizing ChatGPT: here’s how. Nat Biotechnol.

[24] van Dis EAM, Bollen J, Zuidema W, van Rooij R, Bockting CL (2023). ChatGPT: five priorities for research. Nature.

[25] Xue VW, Lei P, Cho WC (2023). The potential impact of ChatGPT in clinical and translational medicine. Clin Transl Med.

[26] Sharma P, Parasa S (2023). ChatGPT and large language models in gastroenterology [published online ahead of print, 2023 May 30]. Nat Rev Gastroenterol Hepatol.

[27] Will ChatGPT transform healthcare? (2023). Nat Med.

[28] Ahn C (2023). Exploring ChatGPT for information of cardiopulmonary resuscitation. Resuscitation.

[29] Howard A, Hope W, Gerada A (2023). ChatGPT and antimicrobial advice: the end of the consulting Infection doctor?. Lancet Infect Dis.

[30] Thorp HH (2023). ChatGPT is fun, but not an author. Science.

